# Rck of *Salmonella* Typhimurium Delays the Host Cell Cycle to Facilitate Bacterial Invasion

**DOI:** 10.3389/fcimb.2020.586934

**Published:** 2020-11-02

**Authors:** Julien Mambu, Emilie Barilleau, Laetitia Fragnet-Trapp, Yves Le Vern, Michel Olivier, Guillaume Sadrin, Olivier Grépinet, Frédéric Taieb, Philippe Velge, Agnès Wiedemann

**Affiliations:** ^1^ INRAE, Université de Tours, ISP, Nouzilly, France; ^2^ IRSD—Institut de Recherche en Santé Digestive, Université́ de Toulouse, INSERM, INRAE, ENVT, UPS, Toulouse, France

**Keywords:** *Salmonella* Typhimurium, cell cycle, DNA damage, checkpoint response, cyclomodulin, invasion

## Abstract

*Salmonella* Typhimurium expresses on its outer membrane the protein Rck which interacts with the epidermal growth factor receptor (EGFR) of the plasma membrane of the targeted host cells. This interaction activates signaling pathways, leading to the internalization of *Salmonella*. Since EGFR plays a key role in cell proliferation, we sought to determine the influence of Rck mediated infection on the host cell cycle. By analyzing the DNA content of uninfected and infected cells using flow cytometry, we showed that the Rck-mediated infection induced a delay in the S-phase (DNA replication phase) of the host cell cycle, independently of bacterial internalization. We also established that this Rck-dependent delay in cell cycle progression was accompanied by an increased level of host DNA double strand breaks and activation of the DNA damage response. Finally, we demonstrated that the S-phase environment facilitated Rck-mediated bacterial internalization. Consequently, our results suggest that Rck can be considered as a cyclomodulin with a genotoxic activity.

## Introduction

Cell proliferation is dictated by the effective progress of the cell cycle into four successive phases: (i) the G1-phase during which cells are synthetizing factors required for DNA replication, (ii) the S-phase, which is the DNA replication phase, (iii) the G2-phase, in which the cells are preparing for the final stage of division, and (iv) mitosis (the M-phase) that generates two identical daughter cells. An exit from the cell cycle can also be observed when cells enter a non-proliferative phase (G0), a state of quiescence (Yang, 2018).

Progression throughout the cell cycle is managed by the specific and sequential expression of cyclin subunits that associate to cyclin-dependent kinase (CDK) and activation (phosphorylation) of CDK, which ensure an ordered succession of the cell cycle phases (Perrot et al., 2018; Martínez-Alonso and Malumbres, 2020). The cyclin D/CDK4 or CDK6 complexes are involved in the progression through the G0/G1-phase; cyclin E/CDK2 in the late G1-phase, which allows the G1-S transition; cyclin A/CDK2 in the early S-phase, which is followed by an accumulation of cyclin A/CDK1 which drives the S/G2 transition and cyclin B-CDK1 promoting the G2/M transition (Malumbres and Barbacid, 2009). Surveillance mechanisms called cell cycle checkpoints are activated at transitions between the cell cycle phases to prevent cells from progressing to the next phase before the prior phase has been correctly completed and to verify faithful DNA replication (S- and G2-phases) and the segregation of chromosomes (M-phase) (Poon, 2016). These checkpoints are orchestrated by upstream damage “sensors” such as ATM and ATR protein kinases. DNA damage, occurring intrinsically upon replication or induced by extrinsic stimulus such as pathogen infections, triggers a checkpoint called DNA damage response (DDR). This will induce an increase in CDK inhibitors (CDKIs) level including p21 (WAF1/Cip1) (Smith et al., 2010) leading to cell cycle arrest and activate the appropriate DNA repair process prior to moving on to the following phase of the cell cycle. In the case of irreversible damage, DDR will eventually initiate apoptosis of the cell (Zhou and Elledge, 2000; Roos and Kaina, 2013; Campos and Clemente-Blanco, 2020).

Many pathogens have developed strategies to control and/or tailor the host cell cycle machinery for their own benefit, allowing their growth and host colonization. The subversion of the cell cycle during virus infections is a widely-known mechanism, allowing an appropriate environment for virus replication and propagation (Fan et al., 2018; Dai et al., 2020). Similarly, bacterial pathogens ability to hijack the host cell cycle have been shown and they have developed cyclomodulins that are able to activate or inhibit eukaryotic cell cycle progression (Nougayrède et al., 2005; Oswald et al., 2005). *Shigella* and pathogenic *Escherichia coli* strains trigger a cell cycle arrest during the G2/M transition (Nougayrède et al., 2001; Iwai et al., 2007), while *Neisseria gonorrhoeae* induce a G1-phase arrest, leading to the inhibition of cell proliferation (Jones et al., 2007). A bacterial infection may not solely influence the cell cycle progression but may also affect the integrity of the DNA of the host cell, activating DDR and cell cycle arrest. Indeed, *Helicobacter pylori* infection stimulates the host cell cycle progression, increasing DNA damage coupled to impaired DNA repair mechanisms and also contributing to genomic instability (Cuevas-Ramos et al., 2010; Toller et al., 2011). The Cytolethal Distending Toxin (CDT) present in many pathogenic strains and the secondary metabolite colibactin produced by strains arboring the *pks* pathogenicity island are prototypical genotoxins that induce DDR-associated cell cycle arrest and genomic instability (Cuevas-Ramos et al., 2010; Taieb et al., 2016). *S.* Typhimurium is a Gram negative enteropathogenic bacterium, responsible for a wide range of food-borne diseases, from gastroenteritis to typhoid fever according to the host (Velge et al., 2012). After oral contamination, the bacterium has developed various mechanisms to enter non-phagocytic cells and to cross the intestinal barrier, which is one of the most crucial steps for *Salmonella* pathogenesis. To date, the most studied invasion system is the type III secretion system-1 (T3SS-1) encoded by the *Salmonella* pathogenicity island-1 (SPI-1) (LaRock et al., 2015). However, a T3SS-1-independent entry system has been characterized, involving the outer membrane protein Rck (Heffernan et al., 1994; Rosselin et al., 2010). Recently, the epidermal growth factor receptor (EGFR) has been identified as a host receptor required for the bacterial invasion process induced by Rck (Wiedemann et al., 2016). The interaction of Rck with the extracellular domain of the EGFR leads to the activation of EGFR, driving a signaling pathway, which allows bacterial internalization. In a physiological context, the epithelial growth factor (EGF) binds to the EGFR. This interaction leads to initiation of numerous signaling pathways, which drives the internalization of the EGF and EGFR complex, as well as proliferation, differentiation and apoptosis of the cells by modifying the expression of many cellular genes (Wee and Wang, 2017). Thus, we hypothesized that the interaction of Rck with the EGFR would not only trigger a signaling cascade enabling bacterial internalization but might also impact other cellular responses such as cell proliferation.

In this study, we showed that Rck can specifically generate host DNA damage modulating the cell cycle to generate a suitable colonization niche for the bacteria.

## Materials and Methods

### Cell Culture and Synchronization

The human intestinal epithelial cell line (HCT116) was grown in McCoy’s 5A medium supplemented with inactivated 10% fetal bovine serum (FBS; Gibco) at 37°C in a humidified atmosphere at 5% CO_2_ (Samba-Louaka et al., 2008). African green monkey fetal kidney epithelial cells (MA104, HPACC: 85102918) were grown in Dulbecco’s modified eagle medium (DMEM), 25 mM glucose supplemented with inactivated 10% FBS and 2 mM L-glutamine (Sigma) at 37°C in a humidified atmosphere at 5% CO_2_.

For cell synchronization, the cells were seeded in 6-well tissue culture plates (5 × 10^5^ cells/well) and grown for 24 h to obtain a 50% confluent cell monolayer. Cells were synchronized in the G0 phase by 24 h serum starvation (Chen et al., 2012). For G1 synchronization, cells were incubated in the presence of 10 µM CDK4/6 inhibitors (Millipore) for 24 h. Cells were blocked at the G1/S phase transition using a double-thymidine block following the protocol of Samba-Louaka et al. (Samba-Louaka et al., 2008). Briefly, cells were incubated in the presence of 2 mM thymidine (Sigma) for 18 h, then washed and cultured for 9 h in normal medium before adding 2 mM thymidine for 16 h. To reversibly block the cells in the S phase, cells were treated with 200 µM azidothymidine (Sigma) for 24 h. For G2-M synchronization, cells were treated for 24 h with 9 µM RO-3306, a CDK1 inhibitor (Millipore), to reversibly block the cells in the G2 phase (Vassilev et al., 2006). As CDK4/6 inhibitors and RO-3306 are dissolved in dimethyl sulfoxide (DMSO), treatment with DMSO alone was used as a control. Cells were released into the cell cycle by replacing the inhibitor-containing medium with non-modified complete cell culture medium.

### Drugs

All drugs were dissolved at the following stock concentrations: cytochalasin D 1 mg/ml (Sigma), caffeine 80 mM (Sigma) and etoposide 100 mM (Sigma). As cytochalasin D and etoposide were dissolved in DMSO, treatment with DMSO was used as a control. The maximal final concentration of DMSO was never superior to 0.1% (v/v) in drug-treated cells.

### Bacterial Strains

The strains and plasmids used in this study are enumerated in **Table 1**. Bacteria were routinely grown in Luria-Bertani (LB) broth with antibiotics at the indicated concentrations: carbenicillin (Cb) 100 µg/ml, chloramphenicol (Cm) 30 µg/ml, and tetracyclin (Tc) 12.5 µg/ml.

**Table 1:** Strains and plasmids used in this study.

Stains	Relevant Characteristic(s)	Source or reference
		
MC1061	*E. coli* hsdR mcrB araD139 Δ(araABC-leu)7679 ΔlacX74 galU galK rpsL thi	Casadaban and Cohen, 1980
*Salmonella* Typhimurium 14028 *Yersinia enterocolitica* **Plasmids**	*S. enterica* subsp. *enterica* ser. Typhimurium wild-type strain, which was isolated from animal tissue *Yersinia enterocolitita* subsp. *enterocolitica* wild-type strain, which was isolated from human tissue.	American Type Culture Collection American Type Culture Collection
pSUP202	Cb^r^, Cm^r^, Tc^r^	Simon et al., 1983 Bio/technology
pSUP202-GFP	Vector carrying the green fluorescence protein (GFP) gene (Cb^r^, Tc^r^)	This study
pSUP202-Rck	Vector carrying the *rck* gene (Cb^r^, Cm^r^)	This study
pSUP202-RckGFP	Vector carrying the GFP and *rck* genes (Cb^r^)	This study
pFPV25.1****	Vector carrying the GFP gene****	Valdivia and Falkow, 1996 ****

### DNA Constructions

The sequences of the primers used are displayed in **Table 2**. The *rck* gene was amplified from *S.* Typhimurium 14028 wild-type strain by PCR using the sense primer *rck* fwd flanked by BamHI restriction site and the reverse primer *rck* rev flanked by SalI restriction site. The *invasin* gene was amplified from *Yersinia enterocolitica* wild-type strain by PCR using the sense primer *invasin* fwd flanked by BamHI restriction site and the reverse primer *invasin* rev flanked by SalI restriction site. The amplified gene was cloned into the pSUP202 expression vector (plasmid) in the cassette encoding for Tc resistance and transformed in *E. coli* MC1061. Using the same technique, the *gfpmut3* gene encoding for the GFP protein was amplified from the pFPV25.1 vector (plasmid) using the sense primer *gfp* fwd carrying a restriction site for EcoRI, BamHI, and XbaI and the reverse primer *gfp* rev flanked by EcoRI restriction site then cloned in the cassette encoding for Cm resistance.

**Table 2:** Primers used in this study.

****Primer name	****Sequence (5’ to 3’)
***rck* fwd** ***rck* rev** ***gfp* fwd** ***gfp* rev** ***invasin* fwd** ***invasin* rev**	CTCGGATCCCTTAACTGTGTTCAGGGAGTTTTATCATG TCTGTCGACTCCCTTTCCTGCTCTCCGTTATC GGGGGGGAATTCGGATCCTCTAGATTTAAGAAG ATGGATGAATTGTACAAATAAGAATTCGGGGGG ATGCACGATATCGGCGTTAATTTATACCTAAGGGGGTAC AGCAGTCGACGCCGCAAGATTGGTATTTAGCACTA

### Adhesion and Invasion Assay

Cells were grown in 24-well plates (Falcon) for 3 days to obtain a confluent monolayer. The cells were infected for 1 h with bacterial suspension in cell medium at a multiplicity of infection (MOI) of 10:1. For the adhesion assay, after bacteria-cell contact, cells were gently washed four times with culture medium and lysed with cold distilled water. The number of total (adherent and intracellular) bacteria was counted after plating serial dilutions on tryptic soy agar (TSA) and the percentage was calculated as the ratio of colony forming units (CFU) of lysates and inoculum. The internalized bacterial level was assessed by a gentamicin protection assay to kill the remaining extracellular bacteria as described previously (Rosselin et al., 2010). The infected cells were incubated in culture medium containing 100 µg/ml gentamicin (Invitrogen) for 90 min and then lysed. The number and the percentage of viable bacteria released from cells were scored and calculated as for the adhesion assay.

### Flow Cytometry and Cell Sorting

The infected cell rate was determined by flow cytometry using an LSRFortessa X-20 analyzer (BD Biosciences, San Jose, CA, USA) based on the green fluorescence expressed by bacteria inside cells. Infected cells with non-fluorescent bacteria were used as a negative control to define the gates corresponding to the cell population with no intracellular bacteria (GFP−), and the one, which contains at least one intracellular bacterium (GFP+). At least 1 × 10^4^ cells were analyzed for each sample using FlowJo software.

To physically separate both cell populations, cell sorting was carried out using a MoFlo Astrios^EQ^ high speed cell sorter (Beckman Coulter Inc, Brea, CA, USA) with the same approach as described previously. Sorted cells were collected in appropriate culture medium containing 10% FBS.

For cell cycle analysis, a cell monolayer was grown in tissue culture dishes (Falcon) for 3 days then infected at a MOI of 100:1 with bacterial suspension for 1 h at 37°C in cell culture medium. After infection, cells were treated with 100 µg/ml gentamicin in cell culture medium containing 10% FBS for 1 h 30 min, then cells were maintained in 10 µg/ml gentamicin in culture medium with 10% FBS for 1 h 30 min [3 h post-infection (p.i.), 4 h 30 min (6 h p.i.), and 22 h 30 min (24 h p.i.)]. Cells were harvested after trypsin treatment. As a control (0 h p.i.), cells were harvested directly after 1 h infection. To analyze the cell cycle, the total mix population and the sorted cells were incubated in a buffer containing 0.01% sodium citrate, 1% Nonidet P-40 (Sigma), 250 µg/ml ribonuclease A (Sigma), and 16 µg/ml propidium iodide (Molecular Probes) to label the nuclear DNA content. The samples were incubated for 30 min at 37°C. Finally, DNA content was analyzed by flow cytometry using an LSRFortessa X-20 analyzer (BD Biosciences) and the percentage of cells in each cell cycle phase was estimated with the Dean Jett-Fox model using FlowJo software. For each sample, 3 × 10^4^ cell events were analyzed. For HCT116 cells, the doubling time is estimated at 15 h. More precisely, the duration of the G1 phase is about 4.1 h, the S-phase 7.1 h, the G2-phase 3.3 h, and the M-phase 30 min (Bernard et al., 2019).

To assess the cell viability and apoptosis, the infected and uninfected cells were trypsined and stained with Annexin V-PE (Invitrogen) and Fixable Viability Dye eFluor780™ (Invitrogen), following the manufacturer’s instructions. The viable cells (Annexin V-PE and FVD- eFluor780™ double negative staining), early apoptotic cells (Annexin V-PE positive and FVD- eFluor780™ negative staining), and necrotic cells (Annexin V-PE and FVD- eFluor780™ double positive staining) were analyzed by flow cytometry using an LSRFortessa X-20 analyzer (BD Biosciences), allowing the analysis of cell death phenomena.

To estimate the level of extracellular EGFR expression at the cell surface, the infected and uninfected cells were fixed by 15 min incubation in PBS with 2% of paraformaldehyde at 4°C. After centrifugation, cells were saturated on ice for 15 min in PBS containing 2.5% BSA and then washed with cold wash buffer (1% BSA in PBS). The monoclonal anti-EGFR (clone 528; Santa Cruz) was diluted to 1:50 using PBS containing 1% BSA and was incubated with cells on ice for 45 min and then washed three times. Alexa 647-conjugated goat anti–mouse antibody (Invitrogen) diluted to 1:200 in PBS containing 1% BSA was used as the secondary antibody and was incubated with cells on ice for 45 min. After three washes, cells were re-suspended in PBS with 2% paraformaldehyde and then the relative fluorescence of the infected and uninfected cells was analyzed using an LSRFortessa X-20 analyzer (BD Biosciences). The relative surface expression of EGFR on the cell surface was considered to be proportional to the mean fluorescence intensity (MFI) and was estimated using FlowJo software. For each sample, 3 × 10^4^ cell events were analyzed.

### H^3^-Thymidine Incorporation Assay

A cell monolayer was grown in tissue culture dishes (Falcon) for 3 days then infected with MC-RckGFP strain at a MOI of 100:1 for 1 h at 37°C in cell culture medium. After the infection, cells were sorted using flow cytometry. GFP− and GFP+ sorted cells were seeded at a density of 1.5 × 10^5^ cells/ml in culture medium containing inactivated 10% FBS, 10 μg/ml gentamicin and H^3^-thymidine at 1 μCi/3.76 × 10^4^ Bq (PerkinElmer). The cells were maintained for 90 min, 3 h, 6 h, or 24 h at 37°C in a humidified atmosphere at 5% CO_2_ followed by scintillation counting (Packard 1600 TR meter, Meriden, CT) (Olivier et al., 2012).

### Alkaline Comet Assay

A cell monolayer was grown in tissue culture dishes (Falcon) for 3 days then infected at a MOI of 100:1 with bacterial suspension for 1 h at 37°C in cell culture medium. After the infection, cells were treated with 100 µg/ml gentamicin in cell culture medium containing 10% FBS for 1 h 30 min then cells were maintained in 10 µg/ml gentamicin in culture medium with 10% FBS for 1 h 30 min (3 h p.i.), 4 h 30 min (6 h p.i.), and 22 h 30 min (24 h p.i.). Cells were harvested after trypsin treatment and sorted using flow cytometry. 2 × 10^5^ GFP− and GFP+ cells were sorted to prepare three slides for comet assays. This assay was carried out exactly as previously described (Trapp-Fragnet et al., 2014). Slides were observed and comet images captured using an Axiovert 200M inverted epifluorescence microscope (Zeiss), coupled to an Axiocam MRm camera (Zeiss). Comet images were analyzed using CometScore software (Tritek). For each slide, 50 cells from different areas were analyzed to calculate the comet parameters (tail extend moment).

### Immunofluorescence Microscopy

HCT116 cell monolayers on 8-well glass bottom iBiDi slides were infected with a bacterial suspension at MOI 100 for 60 min. After incubation at 37°C in a humidified atmosphere at 5% CO2, cells were washed in PBS and fixed for 10 min in 4% paraformaldehyde solution. Then, they were permeabilized for 5 min in 0.2% Triton X-100 in PBS. GFP was stained with a polyclonal antibody GFP Alexa Fluor 488 (Invitrogen; diluted 1:100), and phospho-Histone H2AX (Ser139) was stained with a monoclonal antibody (clone JBW301; Millipore; diluted 1:500). Alexa Fluor 633-labeled goat anti-mouse (Molecular Probes; diluted 1:200) was used as the secondary antibody. Cell nuclei were counterstained with Dapi (Sigma; diluted 1:2,000). The slides were then mounted in fluorescence mounting medium (Dako) and analyzed with an Axiovert 200M inverted epifluorescence microscope (Zeiss), coupled to an Axiocam MRm camera (Zeiss).

### SDS-PAGE and Western Blot

After denaturation in Laemmli sample buffer, proteins were separated by electrophoresis in SDS-polyacrylamide gel and transferred onto a nitrocellulose membrane (BioRad) using Trans-Blot Turbo system (BioRad). Membranes were blocked for 1 h at room temperature and then incubated with primary antibodies overnight at 4°C: cyclin A2 (Cell Signaling, #4656, 1:2,000), cyclin B2 (Santa Cruz, sc-245, 1:1,000), cyclin E1 (Cell Signaling, #4129, 1:1,000), GAPDH (Cell signaling, #2118, 1:1,000) and p21 (Cell Signaling, #2947, 1:1,000). Subsequently, the membranes were washed and incubated with the corresponding anti-mouse/rabbit immunoglobulin G (IgG) horseradish peroxidase (HRP)-conjugated secondary antibodies (anti-mouse IgG, Dako, 1:2,000 and anti-rabbit IgG, Pierce Biotechnology, 1:25,000). Detection was performed by chemiluminescence using a detection kit from Amersham (ECL) and the Fusion FX imaging system (Vilber Lourmat).

### Statistical Analysis

Data were compared using a Mann-Whitney test using GraphPad Prism6.

## Results

### Rck-Mediated Infection Alters the Distribution of Host Cell Cycle Phases

**Figure 1:**
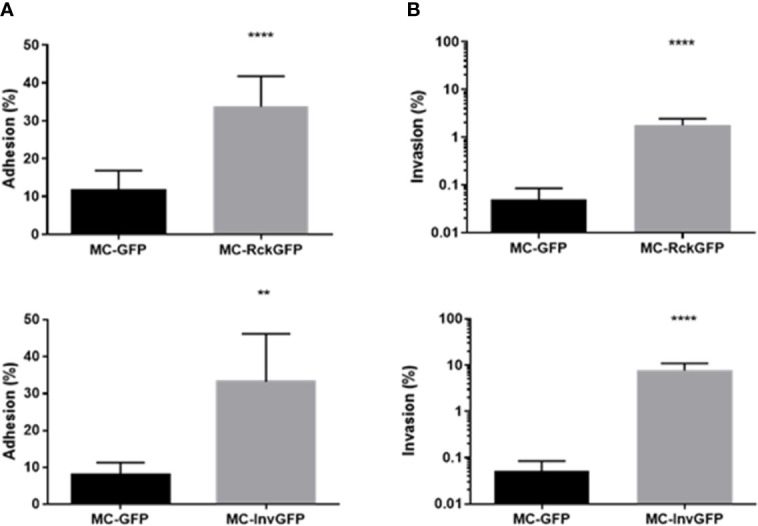
*S.* Typhimurium Rck and *Y. enterocolitica* Invasin are able to induce both adhesion and invasion in intestinal epithelial cells. HCT116 cells were infected with MC-GFP, MC-RckGFP or MC-InvGFP strain for 1 h at 37°C (MOI 10:1). The percentage of total cell-associated **(A)** and intracellular **(B)** bacteria were calculated as the ratio of CFU of cell lysates and inoculum as described in *Materials and Methods*. Results are mean ± SD of results obtained from three independent experiments with two infected wells per experiment. Data were compared using a Mann-Whitney test (**p < 0.01; ****p < 0.0001).

**Figure 2:**
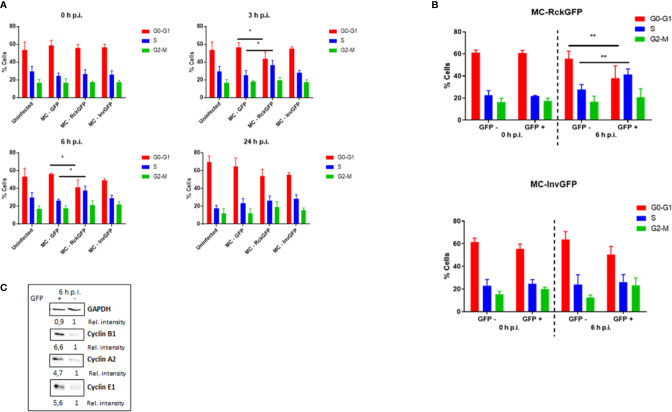
Rck-mediated infection alters the host cell cycle phase distribution. HCT116 cells were infected with MC-RckGFP, MC-InvGFP or MC-GFP strain for 1 h at a MOI of 100. At the indicated times, cell cycle distribution was analyzed **(A)** before or **(B)** after cell sorting [cells with internalized bacteria (GFP+) and cells without internalized bacteria (GFP−)]. Results are means ± SD of the percentage of cells in the different phases of the cell cycle. Data were compared using a Mann-Whitney test (*: p < 0.05; **: p < 0.01; MC-GFP vs. MC-RckGFP for total cells and GFP− vs. GFP+ for sorted cells). At least three independent experiments were performed. **(C)** Cyclin B1, A2 and E1 expression was examined in GFP− and GFP+ cells after sorting at 6 h p.i. and with GAPDH as a protein loading control. This Figure shows an immunoblotting analysis from one experiment, representative of three. *Rel. intensity*, relative intensity.

A previous study of ours revealed that *Salmonella* infection mediated by Rck required an interaction with EGFR on the host cell surface, leading to internalization of the bacteria (Wiedemann et al., 2016). EGFR activation by its natural ligand induces cell proliferation. For that reason, it was of interest to investigate the impact of Rck-mediated infection on the host cell cycle. To investigate overall cellular changes in infected cells without the influence of other *Salmonella* invasion factors, a Rck-mediated invasion model was established. A non-invasive *E. coli* MC1061 strain was used to overexpress Rck of *S.* Typhimurium, as well as the Green Fluorescent Protein (GFP) to identify and sort the infected cells (MC-RckGFP). As an invasion control, an *E. coli* MC1061 strain which overexpresses the *Yersinia enterocolitica* Invasin protein (MC-InvGFP) allowing binding to the beta1 integrin receptor and subsequent invasion into mammalian cells (Isberg and Leong, 1990) was used. First, the ability of MC-RckGFP and MC-InvGFP to bind and invade epithelial intestinal HCT116 cells was compared to the control, *E. coli* MC1061 strain, containing the empty plasmid, which had been used to clone *rck* and *invasin* and which only overexpressed GFP (MC-GFP). As shown in **Figure 1**, the Rck and Invasin-expressing strains were able to bind and invade cells more efficiently than the control MC-GFP (**Figures 1A, B** **)**. These data validate already published results confirming that Rck and invasin are sufficient to induce host cell invasion (Isberg and Leong, 1990; Rosselin et al., 2010). To compare the distribution profiles of the cell cycle phases of infected and uninfected cells, asynchronous HCT116 cells were infected with MC-GFP, MC-RckGFP or MC-InvGFP for 60 min and unbound bacteria were discarded by intensive washing. In order to have enough infected cells to perform the cell cycle analysis cells were infected at MOI of 100 (**Figure S1**). Then, infection was allowed to proceed for 0, 3, 6, or 24 h in the presence of gentamicin to eliminate remaining extracellular bacteria. At different times, the DNA of uninfected and infected cells was dyed with propidium iodide and DNA content was quantified using flow cytometry to determine cell cycle distribution (**Figure S2**). **Figure 2A** displays no significant impairment of the cell cycle phases in uninfected or any of the infected cell populations at 0 h p.i. There was no difference observed at later time points in either uninfected or infected cells with either MC-GFP or MC-InvGFP. However, an increase of about 10% in the percentage of cells in the S-phase and a concomitant reduction in the G0/G1-phase were revealed in cells infected with MC-RckGFP compared to uninfected and MC-GFP infected cells at 3 and 6 h p.i. (**Figure 2A**). At 24 h p.i., the cell cycle profile of Rck-infected cells was similar to that observed in the other conditions, suggesting that Rck-mediated infection induced a transient accumulation of cells in S-phase between 3 and 6 h p.i. Under these experimental conditions, we cannot guarantee that all cells contain at least one intracellular bacterium. Therefore, infected cells were sorted using flow cytometry based on bacterial GFP expression and the cell cycle analysis was focused on infected cells (GFP+) and uninfected bystander cells (GFP−). The GFP+ and GFP− MC-Rck infected cell populations showed a comparable cell cycle profile at 0 h p.i. and no difference was observed between GFP+ and GFP− MC-Inv infected population at 0 or 6 h p.i. In contrast, we evidenced an increase of about 15% in cells in the S phase in the GFP+ MC-Rck infected cell population at 6 h p.i., corroborating the results obtained from the non-sorted cell population (**Figure 2B** and **Figure S3**).

To assess the alteration of the progression of the cell cycle demonstrated by measurement of DNA content, we investigated the expression of cyclins, which are pivotal factors implied in cell cycle regulation. HCT116 cells were infected with MC-RckGFP and the infected (GFP+) and non-infected (GFP−) cells were sorted using flow cytometry at 6 h p.i. The level of cyclin A2, B1, and E1 proteins was evaluated in these two subset populations using Western blot analysis. Consistent with cell cycle observation, infected cells expressed higher protein levels of cyclin A2, B1, and E1 than uninfected cells (**Figure 2C**). Altogether, these results support that Rck-mediated infection induced an alteration of the host cell cycle, leading to an increase in the cell population in the S-phase. This accumulation suggests that the length of the S-phase is extended in response to Rck-mediated infection.

### The Alteration of the Host Cell Cycle Mediated by Rck Does Not Require the Internalization of the Bacterium

**Figure 3:**
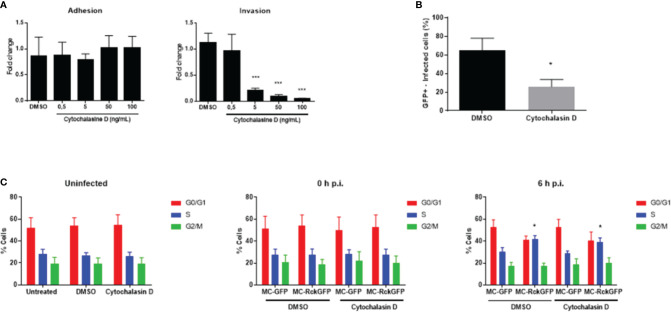
Alteration of the host cell cycle induced by Rck is independent of the complete bacteria internalization process. HCT116 cells were treated with either increasing concentrations of cytochalasin D or DMSO as control before infection with MC-RckGFP. **(A)** Adhesion and invasion levels were then determined and normalized to values obtained with DMSO pretreatment, set at 1. **(B, C)** Cells were treated with cytochalasin D at 100 ng/ml or DMSO for 30 min and then infected with MC-RckGFP with a MOI of 100 for 1 h. **(B)** Following the infection, the percentage of infected cells was determined using flow cytometry. **(C)** At indicated times, cell cycle was analyzed using propidium iodide. Each data represents the mean ± SD of at least three independent experiments. A Mann-Whitney test was used to compare data from the adhesion/invasion assays (***p < 0.001; DMSO vs. Cytochalasin D) and from the flow cytometry experiments, (*p < 0.05; MC-GFP vs. MC-RckGFP).

Rck-mediated internalization requires actin polymerization and rearrangement to allow the engulfment of the bacteria (Rosselin et al., 2010). To evaluate the role of Rck-mediated internalization in the modulation of the host cell cycle, a fungal metabolite that inhibits actin polymerization (cytochalasin D) (Lin and Lin, 1979) was used. First, the cytochalasin D concentration required for inhibiting Rck-mediated internalization in HCT116 cells was determined. As shown in **Figure 3A**, the increasing concentrations of cytochalasin D caused a dose-dependent decline in the number of intracellular bacteria compared to control cells (DMSO treatment). In contrast, no significant differences in the total number of cell-associated bacteria were observed after addition of cytochalasin D at the same concentration range, indicating this treatment does not affect dependent-binding of bacteria to the cell. The highest decrease in internalized bacteria was obtained at 100 ng/ml. Moreover, at this concentration, no cytotoxicity was observed between untreated cells and DMSO-treated or cytochalasin D-treated cells based on morphological changes investigated using flow cytometry (data not shown). Therefore, this concentration was used to study the role of actin polymerization in the host cell alteration induced by Rck. After treatment of cells with 100 ng/ml cytochalasin D or DMSO, the cells were infected with MC-RckGFP. The percentage of infected cells (GFP+) was determined using flow cytometry. As expected, the percentage of MC-RckGFP-infected cells decreased significantly (about 50%) when the cells were incubated with cytochalasin D, compared to DMSO-incubated cells (**Figure 3B**). The DNA content was then analyzed in cells at 6 h p.i. The treatment of uninfected cells with DMSO or cytochalasin D had no effect on the cell cycle distribution (**Figure 3C**). As expected, the percentage of cells in the S-phase was 10% higher in DMSO-treated MC-RckGFP infected cells than in MC-GFP. Interestingly, this higher proportion of cells in the S-phase was still detected in MC-RckGFP infected cells treated with cytochalasin D (**Figure 3C**). These observations suggest that inhibition of Rck-dependent actin polymerization does not mediate cell cycle modulation (accumulation of cells in S-phase). Additionally, our results demonstrate that the internalization of the bacteria is not required for this process.

### Rck-Mediated Infection Induces DNA Damage in Host Cells

**Figure 4:**
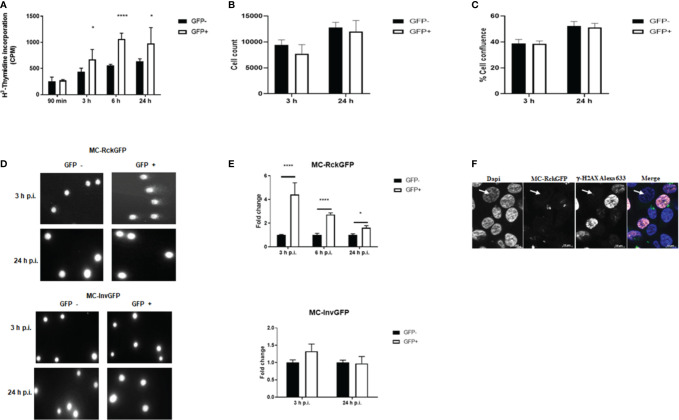
Rck induces host cell DNA damage. HCT116 cells were infected with MC-RckGFP or MC-InvGFP strain. Infected cells (GFP+) and uninfected cells (GFP-) were sorted and then **(A–C)** re-cultivated in presence of H^3^-Thymidine. At the indicated times, the incorporation of H^3^-Thymidine was measured (counts per minute: cpm) **(A)**, the number of cells per well **(B)** and the cell confluence per well were estimated **(C)**. Data show the mean ± SD of one experiment (n = 5), representative of three experiments. **(D, E)** DNA damage assessment was performed using an alkaline comet assay. **(D)** Representative images of comets are shown in GFP− and GFP+ cells at the indicated times. **(E)** For each cell population, the tail extent moment (TEM) parameter was measured with the CometScore software. The data represent the mean ± SD of the fold change of the TEM studied parameters. At least 50 comets were analyzed on three slides/sample over three independent experiments. Data were compared using a t-test (**p < 0.01; ****p < 0.0001; GFP− vs. GFP+). **(F)** Expression and localization of *γ*-H2AX in HCT116 infected with MC-RckGFP. At 6 h p.i., infected cells underwent immunofluorescence staining using a mouse anti-*γ*-H2AX monoclonal antibody and an Alexa Fluor 633-conjugated secondary antibody (shown in red). Nuclei were stained with Dapi (shown in blue), and bacteria expressing GFP (shown in green) were visualized by fluorescence microscopy. A non-infected cell is indicated with a white arrow.

The delay in the host cell cycle in the S-phase induced by Rck, led us to investigate whether DNA synthesis was affected by Rck. A H^3^-thymidine incorporation assay was conducted and at the same time, the number of cells and the cell confluence per cell population has been estimated. HCT116 cells showed a poor cell recovery after cell sorting. Thus, MA104 cells, known to be sensitive to Rck-mediated internalization (Rosselin et al., 2010), were infected with MC-RckGFP and GFP+ and GFP− sorted cells were re-cultured for 90 min, 3 h, 6 h, and 24 h in the presence of H^3^-thymidine. As shown in **Figure 4A**, strikingly, we observed a prompt and higher incorporation (about 1.5-fold) of H^3^-thymidine in the GFP+ infected cell population from 3 until 24 h of re-culture, than in the GFP− infected cells. This result was unexpected since a delay in S-phase (associated to a slower replication rate), would rather lead to a decrease of incorporation of labeled thymidine. However, the number of cells and the cell confluence at 3 and 24 h of re-culture were similar in GFP+ and GFP− cell population (**Figures 4B, C** **)**. As the H^3^-thymidine incorporates into nuclear DNA not only during DNA replication, but also during DNA repair, our results suggest that the delay in the S phase induced by Rck could arise from DNA damage. To assess this hypothesis, we performed a “comet assay”, a test classically used to show the appearance of DNA breaks in the host cell DNA  (Singh et al., 1988). HCT116 cells were infected with either MC-RckGFP or MC-InvGFP as a control and the onset of DNA damage was examined at 3, 6, and 24 h p.i. in GFP+ and GFP− sorted cells using flow cytometry. Representative images of comets obtained from cells infected with MC-InvGFP or MC-RckGFP at 3 and 24 h p.i. are shown in **Figure 4D**. The GFP− and GFP+ cell populations of Invasin infected cells as well as the GFP− cell population of Rck infected cells showed mainly undamaged DNA. However, we clearly observed comets from the GFP+ cell population of Rck infected cells at 3 h p.i., indicating that DNA breaks occurred. In order to quantify the level of damage, the tail extend moment (TEM) parameter was calculated (**Figure 4E**). We could thus show that the TEM was significantly higher in GFP+ Rck infected cells than in GFP− infected cells but tended to decrease during the course of infection (**Figure 4E**). At 3 h p.i., the level of DNA damage was approximately 4.5-fold higher in GFP+ Rck infected cells than GFP− cells, about 2.5-fold higher at 6 h p.i. and decreased to 1.8-fold at 24 h p.i. In order to define the nature of the DNA damage generated in MC-RckGFP-infected cells, we used immunofluorescence staining to monitor the expression and localization of phosphorylated histone H2AX (γ-H2AX), a well-characterized marker of DNA double-strand breaks (DSB) in HCT116 cells (Rogakou et al., 1998). We noticed an overall increase in the fluorescence intensity of the γ-H2AX in the nucleus of MC-RckGFP-infected cells (**Figure 4F**). The staining was visualized either as a pan-nuclear staining indicative of extensive DNA damage or in some infected cells, as a typical punctuated staining reflecting its recruitment to sites of DSB.

These results indicate that Rck-mediated infection induces DSBs. However, the decrease in DNA damage observed during the time of infection suggests either that the damage might be resolved or that damaged cells are eliminated following cell death.

### Rck-Mediated DNA Damage Does Not Lead to Apoptosis in Infected Cells

**Figure 5:**
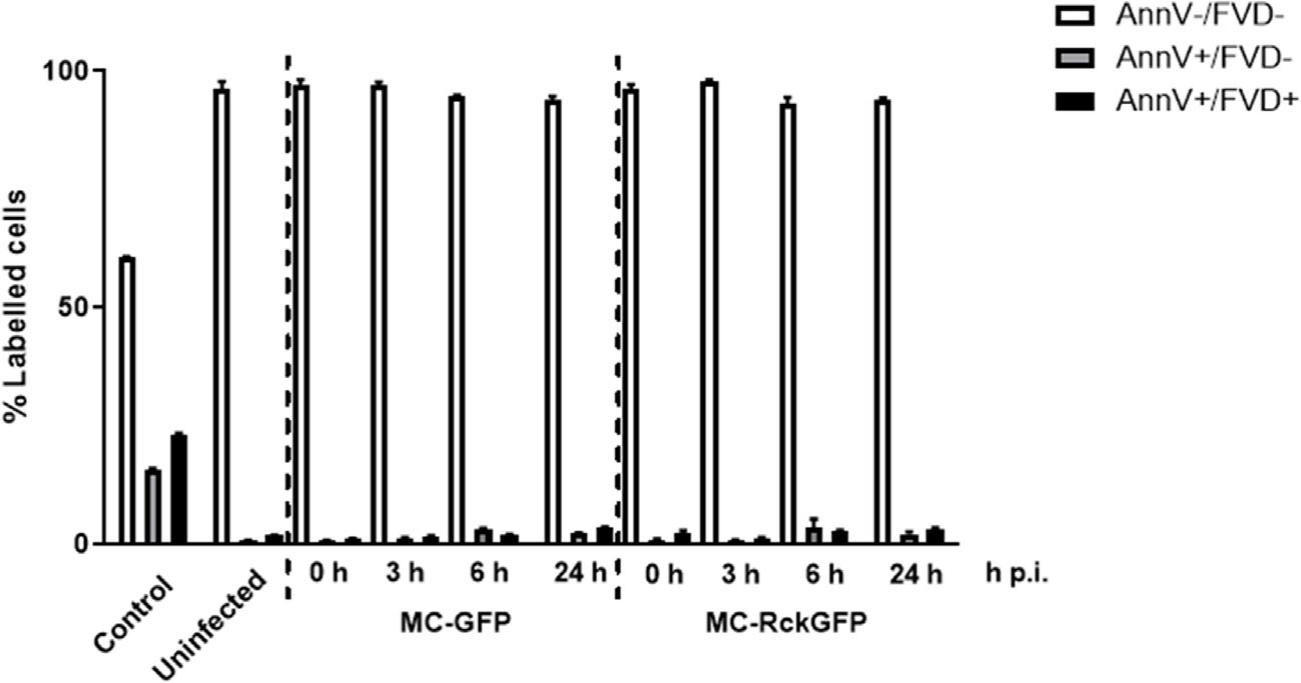
Rck does not induce host cell apoptosis. HCT116 cells were infected with MC-RckGFP or MC-GFP strain for 1 h at a MOI of 100. At the indicated times, a viability assay was performed as reported in *Materials and Methods*. The data represent the mean ± SD of the percentage of labeled cells with Annexin V-PE (AnnV) and FVD-eFluor780 (FVD). At least 20,000 events were analyzed in triplicate over two independent experiments.

To challenge our hypothesis that DNA damage occurring in the MC-RckGFP-infected cells might lead to cell apoptosis, we assayed the cell viability and apoptosis by flow cytometry using a dye exclusion test (indicative of an intact membrane). Infected and non-infected cells were stained with the FVD eFluor780 and Annexin V-PE at 0, 3, 6, and 24 h p.i. Cells were heated and then mixed with living cells to be considered as a positive control. As shown in **Figure 5**, more than 95% of the cells were viable (FVD eFluor780 and Annexin V-PE double negative staining) in infected and uninfected cells. No difference in the percentage of cells in early apoptosis (FVD eFluor780 negative and Annexin V-PE positive staining) or late apoptosis/necrosis (FVD eFluor780 and Annexin V-PE double positive staining) between uninfected and infected cells was detected over the time of infection (**Figure 5**). These results indicate that Rck does not induce cell apoptosis in host cells.

### The DNA Damage Response Is Activated in MC-Rck Infected Cells

**Figure 6:**
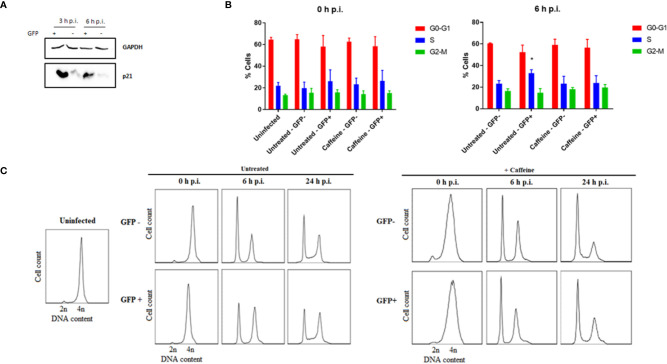
Rck-mediated host cell alteration is a consequence of a DNA damage checkpoint response. HCT116 cells were infected with MC-RckGFP strain for 1 h with a MOI of 100. **(A)** Representative images of immunoblotting analysis corresponding to GAPDH as a protein loading control and p21 expression level of sorted GFP− and GFP+ infected cells at 3 and 6 h p.i. **(B)** DNA histograms generated at 0 and 6 h p.i. of sorted GFP and GFP+ infected cells maintained in the presence of caffeine for 6 h. Results are means ± SD from three independent experiments. Data were compared using a Mann-Whitney test (*p < 0.05; Untreated vs. Caffeine). **(C)** HCT116 cells were treated with RO-3306 for 24 h for G2/M synchronization, then infected with MC-RckGFP strain for 30 min at a MOI of 100. The cells were maintained in the presence or absence of caffeine for 0, 6 and 24 h p.i. GFP+ and GFP− cell populations were sorted by flow cytometry and cell cycle analysis was achieved using flow cytometry. Representative histograms of DNA content for 20,000 events acquired are shown, representative of two independent experiments.

Since the decrease in DNA damage detected at 24 h p.i. in MC-RckGFP-infected cells cannot be elucidated by the stimulation of apoptosis, we tested whether the DDR induced would lead to the eventual reparation of the damage. At first, we sought to determine whether the expression of the p21 protein was affected in infected cells. This multifunctional protein is indeed involved in several cellular processes such as DNA repair, cell cycle arrest, apoptosis and gene transcription triggered notably after DNA damage (Karimian et al., 2016). As DNA breaks were already detected at 3 h p.i. (**Figures 4D, E** **)**, the level of p21 protein was determined at 3 and 6 h p.i. As shown in **Figure 6A**, a strong increase in p21 was detected in the MC-RckGFP-infected cells compared to non-infected cells from 3 h p.i. This result indicates that the delay in the S-phase observed in infected cells is likely mediated by p21 and strongly suggests that DDR pathways are activated in these cells.

To investigate further whether DDR pathways are initiated in MC-RckGFP-infected cells, cells were infected in the presence of caffeine, an inhibitor of ATM and ATR kinases, two key upstream transducers of the DDR (Osman and McCready, 1998; Sarkaria et al., 1999). The cell cycle analysis performed on sorted GFP+ and GFP− MC-Rck infected cells revealed that the accumulation of MC-RckGFP-infected cells in the S-phase was no longer detected in the presence of caffeine (**Figure 6B**). In addition, by a similar approach using cells synchronized in the G2-M phase, we could confirm that the caffeine treatment abolished the delay observed in the untreated GFP+ Rck cell population (**Figure 6C**). Taken together, these data strongly suggest that the modulation of the cell cycle mediated by Rck is dependent on the DDR process and notably the ATR and/or ATM pathways, which are triggered in response to the DNA damage generated in the host DNA upon infection.

### Induction of DNA Damage and the Subsequent S-Phase Delay Facilitates Rck-Mediated Infection

**Figure 7:**
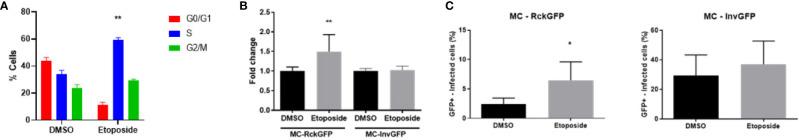
DNA damage and checkpoint responses facilitate Rck-mediated internalization. HCT116 cells were treated for 16 h with either etoposide at 10 µM or DMSO as a control and then infected with MC-RckGFP or MC-Inv GFPGFP strain for 1 h at a MOI of 10. **(A)** Before infection, cell cycle was analyzed on DMSO or etoposide treated cells. Results are means ± SD of the percentage of cells in the different phases of the cell cycle. **(B)** The percentage of internalized bacteria was determined as detailed in Materials and Methods and normalized to values obtained with DMSO pretreatment, set at 1. Results are the mean ± SD obtained from three independent experiments with two infected wells per experiment. **(C)** The percentage of infected cells was determined using flow cytometry according to GFP fluorescence. Results are the mean ± SD obtained from three independent experiments with 30,000 events acquired per experiment. Data were compared using a Mann-Whitney test (*p < 0.05; **p < 0.01; DMSO vs. etoposide).

In order to determine whether the DNA damage and the DDR, notably the S-phase delay can influence Rck-mediated infection, cells were pre-treated (for 16 h) and infected in the presence or absence of etoposide (ETP), a potent inducer of DNA double-strand breaks and S-phase checkpoint activation (Kaufmann, 1998; Bartek and Lukas, 2001; Álvarez-Quilón et al., 2014). We first confirmed that ETP effectively delayed the progression of HCT116 cells into the S-phase. There was a 1.7-fold increase in the number of cells in the S-phase in ETP treated cells compared to untreated cells (**Figure 7A**). ETP-treated cells were infected with MC-RckGFP or MC-InvGFP as a control and the number of internalized bacteria and the percentage of infected cells were quantified. As shown in **Figure 7B**, the percentage of Rck-internalized bacteria was increased 1.5-fold in ETP-treated cells compared to DMSO-treated cells. In contrast, ETP treatment had no effect on the percentage of Invasin-internalized bacteria (**Figure 7B**). In addition, we observed a 2-fold increase in the proportion of cells infected with MC-RckGFP after pre-treatment with ETP, while the ETP treatment had no effect on the proportion of MC-InvGFP infected cells (**Figure 7C**). These data demonstrate that the occurrence of DNA damage and/or the cellular response triggered enhance the Rck internalization process, suggesting that the S-phase environment induced by Rck might facilitate bacterial infection.

**Figure 8:**
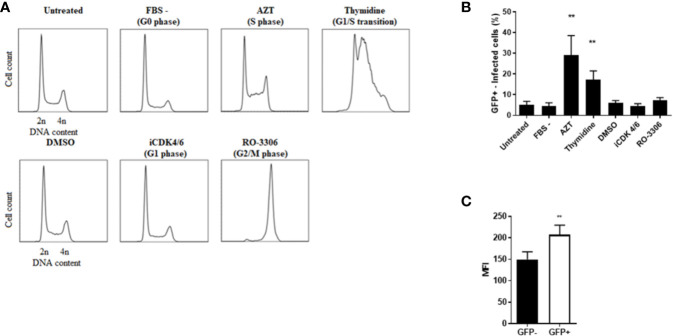
Rck-mediated internalization is facilitated in S-phase cells. HCT116 cells were synchronized at different phases of the cell cycle as indicated. **(A)** Cell cycle was analyzed on synchronized cells using flow cytometry after DNA labelling with propidium iodide. Representative histograms of DNA content for 30,000 events acquired are shown, representative of three independent experiments. **(B)** The synchronized cells were then infected with MC-RckGFP strain for 30 min at a MOI of 10 and the percentage of infected cells was determined according to GFP fluorescence. **(C)** HCT116 cells were infected with MC-RckGFP strain for 1 h at a MOI of 100. At 6 h p.i., the distribution of EGFR was quantified on the cell surface of sorted GFP− and GFP+ infected cells by flow cytometry using a specific anti-EGFR antibody. The MFI was estimated as described in *Materials and Methods*. Results are mean ± SD of results obtained in triplicate from two independent experiments with 30,000 events acquired per experiment. Data were compared using a Mann-Whitney test (**p < 0.01).

We next investigated whether Rck preferentially targeted cells in the S-phase. Cells were synchronized in the G0 phase through 24-h serum starvation (Chen et al., 2012), in the G1 phase using an inhibitor of CDK4/6 (iCDK4/6) treatment, at the G1/S phase transition by a thymidine treatment, in the S phase using azidothymidine (AZT) and in the G2/M phase using the RO-3306, a CDK1 inhibitor (Vassilev et al., 2006). Cells were maintained in the drug-containing medium throughout infection with MC-RckGFP. As a negative control, cells were either cultured in normal medium or in medium supplemented with DMSO. The synchronization of the cells in the expected cell cycle phases was verified using flow cytometry analysis of the DNA content (**Figure 8A**). Then, the percentage of Rck-infected cells (GFP+) was determined by flow cytometry (**Figure 8B**). Compared to the control and other conditions, about 6- and 4-fold more Rck-infected cells were detected after treatment with AZT and thymidine, respectively. This result demonstrates that Rck induces internalization preferentially in the S-phase cells rather than in cells which are in the other phases of the cell cycle.

Rck-mediated internalization requires EGFR (Wiedemann et al., 2016). To verify that the S-phase environment induced by Rck facilitates Rck-mediated internalization, the level of EGFR expression on the cell surface of GFP+ and GFP− Rck infected cell populations was quantified at 6 h p.i. using flow cytometry. **Figure 8C** shows that EGFR was slightly overexpressed in GFP+ Rck infected cells (1.4-fold higher), compared to GFP− Rck infected cells. This result demonstrates that the EGFR expression is induced on the surface of the host infected cell, thus facilitating bacterial infection.

## Discussion

Hijacking checkpoints of the cell cycle is a well-known mechanism used by many pathogens, notably bacteria, to establish infection and growth. In the present study, we showed for the first time that Rck strongly contributes to shaping the host cell cycle in order to favor infection. We showed that infection mediated by *Salmonella* Rck leads to a delay in the cell cycle in the S-phase accompanied by an accumulation of cyclins involved in the progression through the G1 and S phases and of the CKI p21. Many bacteria produce and secrete factors, also called “cyclomodulins”, which are able to alter the host cell cycle and have been introduced as a new class of virulence-associated factors (Nougayrède et al., 2005). Among cyclomodulins, bacterial toxins are potent cell cycle regulators as demonstrated for example for the cytolethal distending toxin (CDT) or the colibactin expressed by *Escherichia coli* which have been demonstrated to induce growth arrest at the G2/M phase (Pérès et al., 1997; Nougayrède et al., 2005; Cuevas-Ramos et al., 2010; reviewed in Taieb et al, 2016). Besides toxins, other bacterial effectors have been identified to interfere with the cell cycle progression and cell proliferation. For example, the type three secreted effector, cycle inhibiting factor (Cif) expressed by enteropathogenic *E. coli*, stabilizes CKI p21 and p27, leading to the inhibition the CDK-cyclin complexes inducing arrest of the cell cycle in G1 and G2 phases (Marchès et al., 2003; Samba-Louaka et al., 2008; Taieb et al., 2011). More recently, it has been pointed out that infection of human epithelial cells with *N. meningitidis* causes an arrest in the G1-phase mediated by the accumulation of p21 and/or p27 (von Papen et al., 2016). Interestingly, brain endothelial cells infected with *N. meningitidis* accumulated in the S-phase and this cell cycle regulation was triggered by the Opa protein and the Opc protein, the major invasins of *N. meningitidis* (Oosthuysen et al., 2016). Therefore, in the light of our results providing evidence for a key role of *Salmonella* Rck in accumulating cells in the S-phase by interfering with major cell cycle modulators, we suggest that Rck belongs to the cyclomodulin family.

As for colibactin or CDT, our results suggest that the cyclomodulin activity of Rck is mediated by an associated genotoxic activity. Indeed, we also established that the S-phase delay induced by Rck cells results from the damage to the genomic DNA in the host and from the subsequent activation of the DDR mediated by ATR and/or ATM pathways. Several studies have shown that pathogenic bacteria are able to provoke an instability of the host cell genome by producing DNA breaks as well as by interacting with the DDR. The involvement of bacterial toxins in generating damage in cellular DNA has been clearly demonstrated. A prominent member of these genotoxins is the colibactin expressed by *E. coli* strains, harboring the *pks* pathogenicity island, which induces host DNA crosslinks and associated DSBs *in vitro* and *in vivo* (Vizcaino and Crawford, 2015; Bossuet-Greif et al., 2018; Wilson et al., 2019). Infection with *Pseudomonas aeruginosa* is also accompanied by the appearance of DSBs induced by the toxin ExoS, and to a lesser extent by ExoT (Elsen et al., 2013). This activity, which is dependent on a functional T3SS on the surface of the bacteria, is associated with ATM activation and could result from the production of reactive oxygen species. The cytolethal distending toxin (CDT) produced by various Gram- pathogenic bacteria including the CDT-related typhoid toxin produced by *S.* Typhi, also shows a strong genotoxic activity. It has indeed been shown that infection with *Salmonella* carrying a functional typhoid toxin results in DSBs (γH2AX foci formation), re-localization of DNA repair proteins (e.g., RPA and NBS1) at the site of DNA damage, the activation of the ATM-Chk2 and ATR-Chk1 signaling pathways, the activation of p53 and synthesis of its transcriptional target p21 and finally the induction of senescence and apoptosis (Weitzman and Weitzman, 2014; Grasso and Frisan, 2015; Taieb et al., 2016; Martin and Frisan, 2020). Here, with an approach using a non-invasive *E. coli* overexpressing Rck of *S.* Typhimurium, we demonstrated that Rck might also play a major role in the DNA damage/DDR induction in infected cells regardless of the expression of the genotoxic typhoid toxin.

One interesting feature of the Rck-induced DNA damage is its reversibility. Indeed, we detected DNA damage at early infection (3 h) and then the level of damage decreased significantly until 24 h. Moreover, our results suggest that apoptosis is not triggered in MC-Rck infected cells. Although we could not show clearly the mechanism involved, we could speculate that the decrease in DNA damage can result from DNA repair mediated through the activation of DDR.

All the activities of Rck described in this study did not require the internalization of the bacterium and could be achieved *via* its interaction with EGFR. Binding of EGF to EGFR expressed at the cell surface induces dimerization of EGFR and subsequent activation of various signal transduction pathways, controlling the internalization of the complex, as well as cell proliferation, differentiation, and apoptosis. Recently, EGFR has been identified as the receptor of the *Salmonella* invasion protein Rck, whose interaction leads to the activation of a signaling cascade leading to bacterial internalization (Wiedemann et al., 2016). A few *in vivo* and *in vitro* studies have highlighted interplay between *S.* Typhimurium and cell proliferation by targeting the host cell cycle (Naughton et al., 1995; Maudet et al., 2014). Thus, it is likely that the interaction of Rck with EGFR does not only induce an internalization mechanism but also modulates other cellular responses. To our knowledge, the tyrosine kinase activity of EGFR affects a signaling cascade involved in the DNA damage response and/or DNA repair (Dittmann et al., 2008; Bai et al., 2012; Karimian et al., 2019). In addition, EGFR signaling has been shown to impact the phosphorylation level of some DNA repair proteins, e.g., DNA-PKcs, ATM and H2AX, which all require phosphorylation at specific sites for activation (Meyn et al., 2009). Remarkably, consistent with these activities, we showed that these signaling proteins are also implicated in cellular response to Rck-mediated infection. The fact that EGFR seems to be implicated in tumor resistance to chemo/radiotherapy *via* activation of DNA DSB repair pathways such as non-homologous end joining (NHEJ) mechanism might also explain the reversibility of Rck-induced DNA-damage (Rodemann et al., 2007).

Several strategies have been developed by bacterial pathogens to promote their colonization by controlling the host cell cycle. The concept that cell cycle modulation might promote bacterial colonization has been demonstrated for *Shigella* and for *Helicobacter pylori* that have adapted to the hostile conditions found at the gastric mucosal surface (Iwai et al., 2007; Mimuro et al., 2007). In addition, infection by *Listeria monocytogenes* induces an S-phase delay of the host infected cells to promote bacterial internalization (Leitao et al., 2014). In the case of *Staphylococcus aureus*, the infection induces a delay in the G2/M transition, favoring increased bacterial internalization and enhanced intracellular proliferation (Alekseeva et al., 2013; Deplanche et al., 2015). Similar to *Staphylococcus aureus*, *Bordetella* species and *Bacillus anthracis* alter the host cell cycle to promote their internalization in non-phagocytic cells (Martín et al., 2015). Concerning *S.* Typhimurium, previous research has shown that in epithelial cells, *S.* Typhimurium infection blocks cell cycle in the G2/M phase (Maudet et al., 2014), which facilitates *Salmonell*a infection, as *Salmonella* preferentially invades mitotic cells (Santos et al., 2013). Our data do not support this idea. One explanation could be that the *in vitro* conditions in which the *Salmonella* strains used in this study were grown, did not allow Rck to be expressed efficiently (Kim and Surette, 2006; Rosselin et al., 2010). In our study, a non-invasive *E. coli* strain overexpressing Rck was used to study precisely the impact of this outer membrane protein on the host cell cycle in the absence of known and unknown invasion factors expressed by *Salmonella* (Rosselin et al., 2011; Roche et al., 2018).

The intestinal epithelium undergoes constant cell renewal in which cell production balances cell loss. To achieve colonization, bacteria have to impede epithelial cell turnover. Our results suggest that Rck could hinder the progression of the host cell cycle, increasing the duration of its S-phase to facilitate bacterial infection, and thus creating a suitable colonization niche. In *Salmonella* pathogenicity, the importance of the interaction of Rck with EGFR is still undefined. It has been shown that quorum sensing regulates Rck expression, involving SdiA in an N-acylhomoserine lactone–dependent manner (Smith and Ahmer, 2003; Abed et al., 2014), suggesting an intestinal role of Rck. Furthermore, in a murine *in vivo* model, a role in the fitness of *Salmonella* has been demonstrated for Rck, thus reinforcing this hypothesis (Dyszel et al., 2010). In intestinal proliferative cells, intestinal stem and transit amplifying cells, EGFR is expressed on the luminal, apical side of the cells. *S.* Typhimurium has access to the epithelial crypts in the mouse model of infection, allowing the bacteria to invade, persist and survive in the intestinal epithelium (Müller et al., 2012). We may thus hypothesize that luminal bacteria could directly interact and infect these cells *via* Rck to delay the progression of the cell cycle, leading to reduced intestinal cell renewal and exfoliation, thus impairing intestinal function. This hypothesis is currently being investigated in our laboratory by using *Salmonella* infected gastrointestinal organoid models.

In conclusion, our results shed new light on the function of Rck which should be considered both as an invasin and as a “cyclomodulin” affecting the cell cycle machinery and with a further role as a “genotoxin” which alters the DNA integrity of epithelial cells. Undeniably, our findings contribute to detail further the mechanisms underlying microbial pathogenesis and to enhance understanding of how *Salmonella* colonizes the intestine.

## Data Availability Statement

All datasets presented in this study are included in the article/**Supplementary Material**.

## Author Contributions

AW designed the research. JM, EB, LF-T, YV, MO, GS, and AW carried out research. OG and FT contributed new reagents and analytic tools. JM, EB, LF-T, and AW analyzed data. LF-T and AW wrote the manuscript. PV provided critical comments. All authors contributed to the article and approved the submitted version.

## Funding

This work was supported by the ERA-NET InfectERA (Sal Host Trop, ANR-15-IFEC-0003). JM holds a doctoral fellowship granted by the Ministère de l’Enseignement Supérieur, de la Recherche et de l’Innovation.

## Conflict of Interest

The authors declare that the research was conducted in the absence of any commercial or financial relationships that could be construed as a potential conflict of interest.

## Supplementary Material

Supplementary Figure 1 Percentage of infected cells based on GFP fluorescence. HCT116 cells were infected with either MC-Rck, MC-GFP, or MC-RckGFP strain for 1 h at 37°C (MOI of 100). **(A)** The percentage of infected cells was determined using flow cytometry based on bacterial GFP expression. Cells infected with non-fluorescent MC-Rck strain were used to define the region corresponding to the natural basal autofluorescence. **(B)** The percentage of infected cells was investigated at the indicated times after infection with either MC-GFP or MC-RckGFP.

Supplementary Figure 2 Illustration of the gating strategy to analyze the cell cycle of infected cells. HCT116 cells were infected with either MC-GFP or MC-RckGFP strain for 1 h at 37°C (MOI 100:1). At 6 h p.i., cells were lysed and stained with propidium iodide. Samples were analyzed by flow cytometry using a gating strategy to exclude: (i) the cell debris using side scatter (SSC-A) versus forward scatter (FSC-A). This allows to identify cells of interest based on size and granularity (Region 1); (ii) doublets and aggregates (Region 2) using propidium iodide fluorescence width (PI-W) versus area (PI-A) on the cell population of the Region 1 to select single cells (Region 2); (ii) the doublets and aggregates using propidium iodide fluorescence height (PI-H) versus area (PI-A) on the cell population of the Region 1 to select single cells. Single cell region (Region 1 and Region 2 and Region 3) is then displayed as a histogram using PI-A parameter. Three populations result from the histogram: two Gaussian curves (2n and 4n DNA peaks) and the S-phase population. Neighboring populations overlap each other. In consequence, the Dean Jett-Fox model from FlowJo software was used to de-convolute the populations and set percentage values to each population (G0/G1 is shown in red, S in blue and G2/M in green).

Supplementary Figure 3 Representative DNA histograms from Rck-infected and uninfected cells. HCT116 cells were infected with either MC-RckGFP or MC-GFP strain for 1 h (MOI of 100). At the indicated times, the DNA content was analyzed for 20,000 non-sorted **(A)** or sorted (cells with internalized bacteria (GFP+) and cells without internalized bacteria (GFP-) **(B)** events using flow cytometry.

## Abbreviations

ATM, ataxia-telangiectasia mutated; ATR, ATM and Rad3-related protein; AZT, azidothymidine; Cb, carbenicillin; CDK, cyclin-dependent kinase; CDKI, CDK inhibitor; CDT, cytolethal distending toxin; Cm, chloramphenicol; cpm, counts per minute; DDR, DNA damage response; DSB, double strand breaks; DMSO, dimethyl sulphoxide; EGF, epithelial growth factor; EGFR, epidermal growth factor receptor; ETP, etoposide; FBS, fetal bovine serum; GFP, green fluorescent protein; LB, Luria-Bertani; MFI, mean fluorescence intensity; MOI, multiplicity of infection; p.i., post-infection; PI3K, phosphatidylinositol-3-kinase; Rck, resistance to complement killing; SPI-1, *Salmonella* pathogenicity island-1; Tc, tetracyclin; TEM, tail extend moment; T3SS-1, type III secretion system-1; TSA, tryptic soy agar.
