# Supplementary figures and images for: Rck of Salmonella Typhimurium Delays the Host Cell Cycle to Facilitate Bacterial Invasion
Source: Front Cell Infect Microbiol. 2020 Nov 2;10:586934. doi: 10.3389/fcimb.2020.586934 (PMC7734966; doi:10.3389/fcimb.2020.586934)

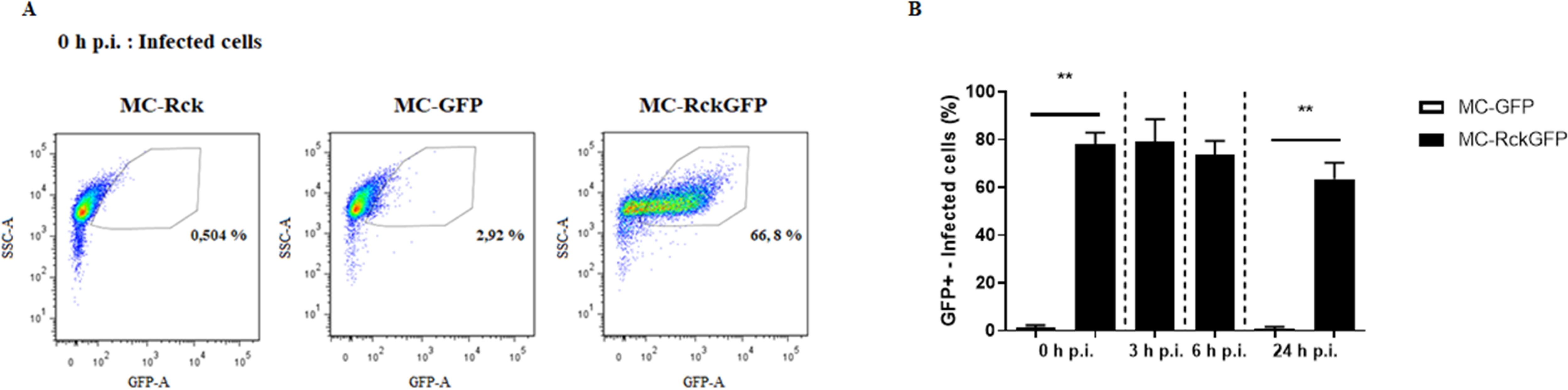

Supplement: Supplementary Figure 1 — Percentage of infected cells based on GFP fluorescence. HCT116 cells were infected with either MC-Rck, MC-GFP, or MC-RckGFP strain for 1 h at 37°C (MOI of 100). (A) The percentage of infected cells was determined using flow cytometry based on bacterial GFP expression. Cells infected with non-fluorescent MC-Rck strain were used to define the region corresponding to the natural basal autofluorescence. (B) The percentage of infected cells was investigated at the indicated times after infection with either MC-GFP or MC-RckGFP. [file Image_1.tif]

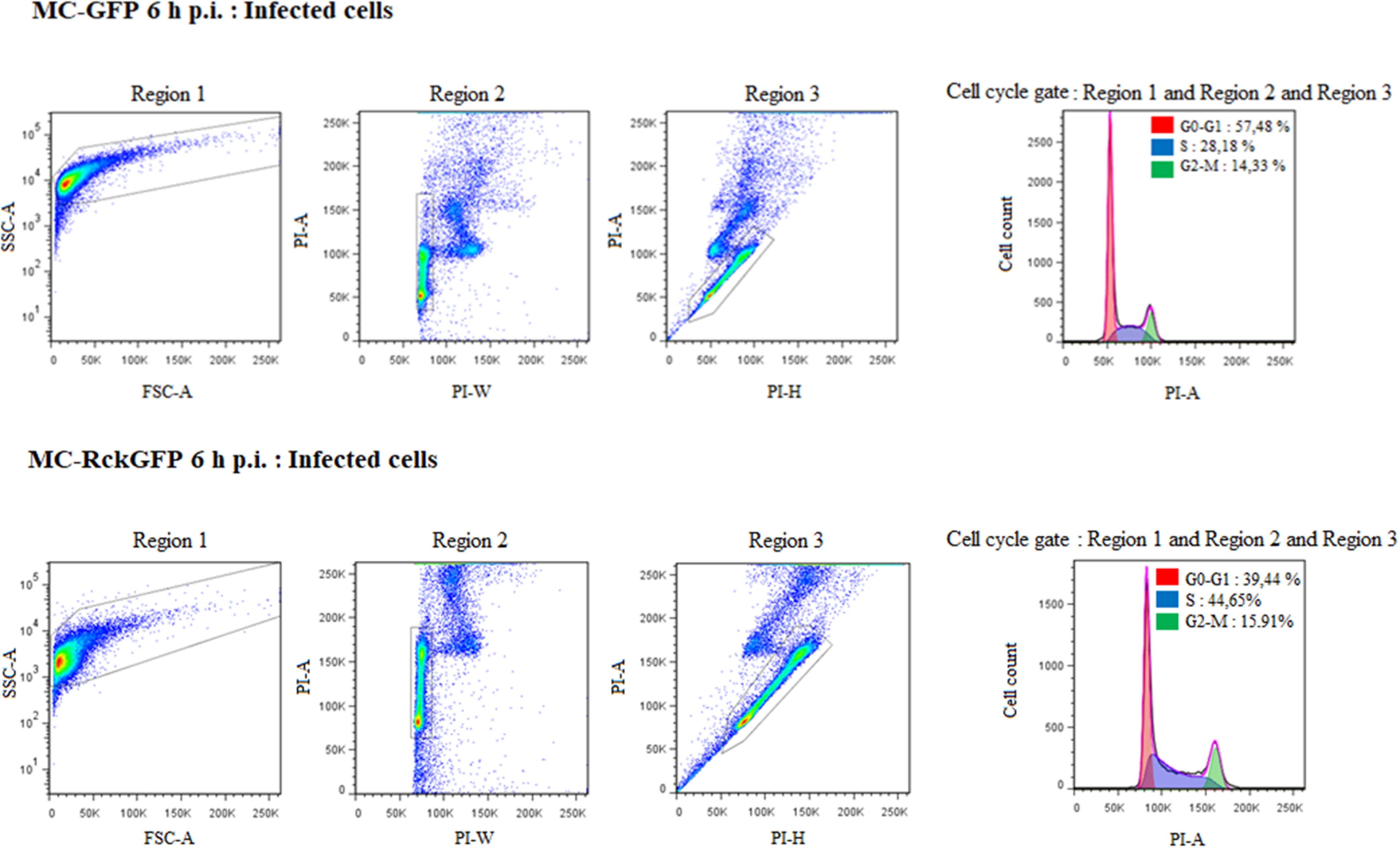

Supplement: Supplementary Figure 2 — Illustration of the gating strategy to analyze the cell cycle of infected cells. HCT116 cells were infected with either MC-GFP or MC-RckGFP strain for 1 h at 37°C (MOI 100:1). At 6 h p.i., cells were lysed and stained with propidium iodide. Samples were analyzed by flow cytometry using a gating strategy to exclude: (i) the cell debris using side scatter (SSC-A) versus forward scatter (FSC-A). This allows to identify cells of interest based on size and granularity (Region 1); (ii) doublets and aggregates (Region 2) using propidium iodide fluorescence width (PI-W) versus area (PI-A) on the cell population of the Region 1 to select single cells (Region 2); (ii) the doublets and aggregates using propidium iodide fluorescence height (PI-H) versus area (PI-A) on the cell population of the Region 1 to select single cells. Single cell region (Region 1 and Region 2 and Region 3) is then displayed as a histogram using PI-A parameter. Three populations result from the histogram: two Gaussian curves (2n and 4n DNA peaks) and the S-phase population. Neighboring populations overlap each other. In consequence, the Dean Jett-Fox model from FlowJo software was used to de-convolute the populations and set percentage values to each population (G0/G1 is shown in red, S in blue and G2/M in green). [file Image_2.tif]

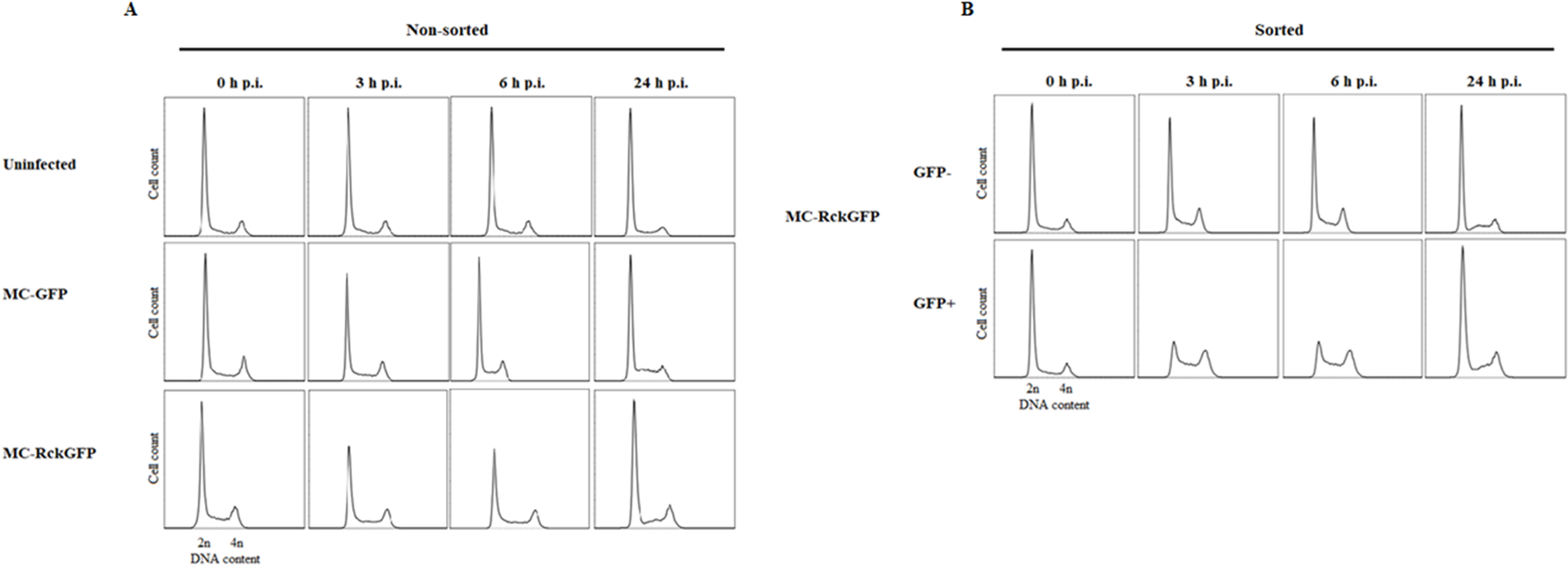

Supplement: Supplementary Figure 3 — Representative DNA histograms from Rck-infected and uninfected cells. HCT116 cells were infected with either MC-RckGFP or MC-GFP strain for 1 h (MOI of 100). At the indicated times, the DNA content was analyzed for 20,000 non-sorted (A) or sorted (cells with internalized bacteria (GFP+) and cells without internalized bacteria (GFP-) (B) events using flow cytometry. [file Image_3.tif]
